# Supplementary material for: Test-retest reliability of the Performance of Upper Limb (PUL) module for muscular dystrophy patients
Source: PLoS One. 2020 Sep 28;15(9):e0239064. doi: 10.1371/journal.pone.0239064 (PMC7521751; doi:10.1371/journal.pone.0239064)
Supplement: Attachment — Submitted filename: Response to Reviewers.docx [file pone.0239064.s003.docx]

Dear Editor and Reviewers,

Thank you for your careful analysis of our manuscript, and for allowing us to address the reviewers' minor comments.

We have greatly appreciated the comments and the suggestion provided. We are glad that the Reviewers have appreciated our study..

We are uploading our point-by-point response to the comments (below), the manuscript with highlighted changes with respect to the previous version (in red), and the updated manuscript.

Note: For the ease of tracking, we use the following color-coding for the three categories demanded in the response letter:

a) **"Reviewer's concerns"** are given in Black-Color text

b) "**Author responses** (responses to the concern)" are given in Blue-Color text

c) "**Author actions** (actions to remedy the concerns)" are given in Red-Color text

**Reviewer #1:**

Reviewer #1: I appreciate the authors addressing all of the reviewers’ previous comments. The changes have improved the manuscript clarity and detail. Just a few minor comments that would benefit from being addressed are included below. All other recommendations were addressed. No need for further peer review if the suggestions below are addressed.

• Minor grammatical errors remain throughout, though most have been corrected. For example, the final sentence of the abstract is a run-on sentence.

We have revised language, and we corrected the final sentence of the abstract. In addition, we have sent the manuscript to a mother tongue colleague in USA, who performed a thorough English grammar review.

• In the introduction and discussion, the authors state that the PUL can evaluate ambulatory individuals without floor or ceiling effects. I feel this statement is too strong as Pane et al 2014 demonstrated that quite a few ambulatory boys had ceiling effects on the PUL, especially if they were still strong ambulators. (“The 6 Minute Walk Test and Performance of Upper Limb in Ambulant DMD Boys”).

We have updated the sentence in the Introduction section as follows:

“It records the minimum variations in functionality, limiting ceiling and floor effects, through its scoring system (Mercuri et al., 2011), even if some ceiling effect has been observed in ambulant DMD boys (Pane et al., 2014).”

Instead, we removed the comment in the Discussion section, since it was already presented in the Introduction section.

• It seems that perhaps the data in figure 1 and 2 from PUL1 and PUL 2 are swapped (or they are reported backwards in the text). In the text it says the min score from PUL1 was 17 and min from PUL2 was 19, but in Fig 1, the lowest symbol is definitely lower in PUL2. I think this is the same problem as in Fig 2.

We thank the reviewer for spotting this out. We updated both figure 1 and 2.

• Would be nice to have a SD for the CVs. Also, there was still an n=9 for CV for the shoulder level subgroup analysis, and you report a CV for the BMD subgroup analysis with an n=6. I feel the CV for the shoulder dimension should be reported as the reason given for not reporting it is not valid.

As suggested, we added the SD for the CV for the entire population, as well as for the subgroup analyses. Moreover, we added the computation of the CV for the shoulder level, specifying that it is related to a limited number of patients - those who were able to perform the related items.

• It would be nice to point out in the discussion that the MDC in DMD was 5 rather than 4 as it was for the entire cohort and BMD/LGMD.

Thanks for highlighting this observation. We have included the following sentence in the Discussion section:

“It is worth a note that for DMD population only, MDC resulted to be equals to 5 points. This observation should be considered if only DMD patients are recruited, or for single-patient longitudinal evaluation.”

• The following sentence in the conclusion should be toned down a bit: “Thanks to this novel study, the use of the PUL module can be extended to clinical trials involving BMD and LGMD, and not only DMD.” I think this study lays the groundwork for the inclusion of the PUL in other muscular dystrophies, but as you mention, further validation is definitely required.

As suggested, we modified the sentence in the conclusion section as follows:

“Moreover, this novel study lays the groundwork for the inclusion of the PUL module in other muscular dystrophies, and not only in DMD.”

**Reviewer #2:**

My previous concerns have been thoughtfully and thoroughly addressed. The paper will be a value to the field.

We thank the reviewer for appreciating our work, and for the contribution with previsous revisions.
